# Automated manufacturing process for sustainable prototyping of nuclear magnetic resonance transceivers

**DOI:** 10.5194/mr-6-199-2025

**Published:** 2025-07-29

**Authors:** Sagar Wadhwa, Nan Wang, Klaus-Martin Reichert, Manuel Butzer, Omar Nassar, Mazin Jouda, Jan G. Korvink, Ulrich Gengenbach, Dario Mager, Martin Ungerer

**Affiliations:** 1 Institute of Microstructure Technology (IMT), Karlsruhe Institute of Technology (KIT), Hermann-von-Helmholtz-Platz 1, 76344 Eggenstein-Leopoldshafen, Germany; 2 Voxalytic GmbH, Rosengarten 3, 76228 Karlsruhe, Germany; 3 Institute for Automation and Applied Informatics (IAI), Karlsruhe Institute of Technology (KIT), Hermann-von-Helmholtz-Platz 1, 76344 Eggenstein-Leopoldshafen, Germany

## Abstract

Additive manufacturing has enabled rapid prototyping of components with minimum investment in specific fabrication infrastructure. These tools allow for a fast iteration from design to functional prototypes within days or even hours. Such prototyping technologies exist in many fields, including three-dimensional mechanical components and printed electric circuit boards (PCBs) for electrical connectivity, to mention two. In the case of nuclear magnetic resonance (NMR) spectroscopy, one needs the combination of both fields; we need to fabricate three-dimensional electrically conductive tracks as coils that are wrapped around a sample container. Fabricating such structures is difficult (e.g., six-axis micro-milling) or simply not possible with conventional methods. In this paper, we modified an additive manufacturing method that is based on the extrusion of conductive ink to fast-prototype solenoidal coil designs for NMR. These NMR coils need to be as close to the sample as possible and, by their shape, have specific inductive values. The performance of the designs was first investigated using electromagnetic field simulations and circuit simulations. The coil found to have optimal parameters for NMR was fabricated by extrusion printing, and its performance was tested in a 1.05 T imaging magnet. The objective is to demonstrate reproducible rapid prototyping of complicated designs with high precision that, as a side effect, hardly produces material waste during production.

## 1 Introduction

Additive manufacturing of electrical tracks with widths in the range of tens of microns, in both 2D and 3D, and providing low electrical resistances is of great interest for electronic applications such as interconnects, wire bonds, sensors, antennas, transistors, and transparent electrode grids [Bibr bib1.bibx24].

Low-viscosity inks ( <20mPas ), as frequently used in ink jet printing, are highly susceptible to surface forces and, therefore, tend to develop irregular structural topographies, especially with respect to nonuniform distributions of the ink [Bibr bib1.bibx17]. The aspect ratio of a printed line describes the quotient of layer thickness and width. This ratio should be as high as possible in order to achieve sufficient conductivity when miniaturizing the line width. Typical aspect ratios of single-layer (single-pass) ink-jet-printed conductor tracks, for example, are often less than 0.01 [Bibr bib1.bibx28]. High-viscosity inks (in general pastes) allow for higher solid loading, which leads to lower resistances (20 %–70 % of the bulk conductivity), and are also less prone to irregular topographies. These inks allow much higher aspect ratios, e.g., up to 0.14 for silver conductors on polyimide [Bibr bib1.bibx29]. In the class of digital printing processes, the extrusion-printing (EP) process (contact dispensing) enables printing of inks with the highest viscosities [Bibr bib1.bibx17]. Extrusion printing is based on the ink transfer from the printing nozzle (dispensing tip) to the substrate by means of a continuous fluidic bridge forming a fluid filament during relative motion between the nozzle and the substrate [Bibr bib1.bibx29]. The force for generating the fluidic bridge and for maintaining the appropriate ink mass flow during printing is applied to the ink by means of dispensing principles, e.g., time pressure, positive displacement (piston), or a rotary screw (Auger pump) [Bibr bib1.bibx17]. [Bibr bib1.bibx29] developed a versatile and modular EP system based on the time–pressure principle and tested it for various screen-printing inks and printing nozzle types. [Bibr bib1.bibx3] applied this printing principle for the fabrication of radio frequency (RF) transmission lines for photonic integrated circuits. They pointed out the robustness of the process in relation to substrate properties and the far better line edge quality on ceramic substrates in contrast to transmission lines that were ink jet printed with low-viscosity inks. The extrusion-printing technology and the modular system presented by [Bibr bib1.bibx29] show great potential to fabricate nuclear magnetic resonance (NMR) coils and to directly print complicated coil geometries on a glass tube. This helps in improving the filling factor of the coil and allows the use of different sample geometries based on the requirements of the measurements.

NMR is one of the most specific spectroscopy techniques available and is able to elucidate structures down to the atomic level. However, the acquisition of a well-resolved spectrum depends on several factors, such as the homogeneity of the static magnetic field, which needs to be in the range of parts per billion, achieved by the minimization of the magnetic susceptibility mismatch caused by material jumps in the vicinity of the detector. The resonator coil must be properly tuned and matched, and a sufficient SNR (signal-to-noise ratio) must be attained. Field homogeneity corrections can be achieved with shimming coils and typically depend strongly on the spectroscopist's experience. The simplest coil types are solenoids, but there are much more sophisticated coil designs that differ in terms of signal quality and signal-to-noise ratio (SNR). Consider the equation of the SNR by [Bibr bib1.bibx12]:

1 $$\text{SNR}\propto \frac{kV_{s}B_{1}}{\sqrt{R}},$$

where $V_{s}$ is the volume of the sample, k is the filling factor of the coil, $B_{1}$ is the magnetic field produced by the coil, and R is the resistance of the coil. As the SNR of the coil also depends on the volume of the sample and the filling factor, a low volume results in a lower SNR value. However, this effect can be compensated for by improving the filling factor of the coil, which helps to improve the SNR, especially of micro-coils, where not all parts can be shrunk equally. To get a decent filling factor of the coil, the conductive part responsible for generating the radio frequency (RF) magnetic field must be as close as possible to the sample [Bibr bib1.bibx15]. However, the size of the coil and the length of the conductive track also affect the coil's self-resonance. If the self-resonance is below the nucleus's Larmor frequency, it cannot be tuned and matched to that frequency using capacitors, and if the self-resonance is too high, the tuning and matching to the lower frequency are limited by the available capacitance values [Bibr bib1.bibx12]. Hence, the coil needs to be designed such that it both fulfills the requirement of the filling factor and, at the same time, can be tuned and matched to the target frequencies. Coils are normally simulated before fabrication, but in the case of micro-coils, the conventional fabrication methods have significant tolerances, which lead to large variations from coil to coil. In this work, we present a routine which eliminates the need for repeated prototyping to get dedicated coil parameters. The coil design and fabrication cycle starts with the development of a three-dimensional solenoid geometry model, which is used to compute the RF magnetic field radiation and the S parameters (reflection and transmission coefficients) at the termination port of the coil using COMSOL AB. The S parameters are used to calculate the coil's electrical properties such as self-resonance of the coil, lumped component values required for tuning and matching at the target frequency, and the quality factor at the target frequency using the Advanced Design System (ADS) from Keysight Technologies. After investigating all of the parameters, the coil which best meets the requirements is manufactured using the extrusion-printing technology. The performance of the coil was tested by acquiring NMR spectrum and MR images. The objective is to make the method easily scalable for industrial applications with a sustainable method, as was shown by [Bibr bib1.bibx21], as developing suitable micro-coils can lead to several iterations before a functional design is found. The idea to make micro-MR coils [Bibr bib1.bibx23] in a non-manual way has been around for quite a while, like in the publications of [Bibr bib1.bibx25], [Bibr bib1.bibx20], [Bibr bib1.bibx4], and [Bibr bib1.bibx10]. In this paper, we plot the full route from the planning of a coil with desired properties to its manufacturing and testing. One may question the requirement of a new fabrication technology given the fact that conventional solenoidal micro-coils can be hand-wound [Bibr bib1.bibx23], yielding excellent results. One reason is that there are other coil designs apart from just solenoids, e.g., Helmholtz, birdcage, or saddle coils, to mention a few [Bibr bib1.bibx11]. These designs cannot be manufactured manually at the micro-scale. The second reason for direct coil writing is the improved precision. Every material change next to the sample causes disturbances in the magnetic field homogeneity; this is a particular problem for materials that need to be close to the sample, like the micro-coil itself. Therefore, it is good to know these disturbances upfront so that they can be minimized by design, reducing the magnetic field disturbances that need to be corrected by pre-disturbing the field (so-called shimming). Also, the electrical tuning and matching get easier if the coils show small part-to-part variations. The gain that comes from the high geometrical precision is a little bit reduced by the reduced conductivity of sintered metal tracks compared to bulk wires. The sintered tracks show about 20 %–70 % of the bulk conductivity, leading to a slightly higher electrical noise.

## 2 From simulation-based design to specific coils

The closer the coil is to the sample, the better the filling factor and the subsequent improvement in SNR are, as described in Eq. ([Disp-formula Ch1.E1]). The smaller size of the glass tube requires coils in the same dimensional range, which results in higher self-resonance. Though higher self-resonance can be tuned and matched to lower target frequencies, the tuning range is limited by the available capacitance values. Another factor that plays an important role is the fact that, by shrinking the coil, the conductive-track diameters are also reduced in order to get well-defined geometries, but these thinner tracks lead to higher resistance of the coil since the cross-sectional area of the conductor is lower [Bibr bib1.bibx15]. The increase in resistance raises the thermal noise of the coil and reduces its RF efficiency ( $B_{1}/\sqrt{W}$ ). However, to significantly increase the noise, the temperature has to increase a lot, such that this effect is not dramatic. On the other hand, the lower the resistance, the better the RF efficiency.

To find the perfect geometry which meets all the requirements, a computational domain was set up to simulate the coils' RF behavior. For the computation, various solenoid geometries were investigated, where the diameters of the coils were kept constant at 1.5 mm , and the number of turns N and the pitch p , i.e., the distance between two turns, were varied. For the simulation, the sample material chosen was $H_{2}O$ , where the sample volume was approximated by a cylindrical geometry of height 1 mm and diameter 1 mm . The glass tube was neglected in the simulation since it would increase the complexity of the calculation without having a major effect on the result due to the low dielectric constant. The sample volume was kept constant for different coil geometries to simulate the uniform influence of the sample on the coil properties. The computation of the model was performed using the RF module from COMSOL AB. The computational geometries and the truncation of the boundaries are described in Fig. [Fig F1]a. Mathematically, the [Bibr bib1.bibx8] model is summarized as follows:

2∇×(∇×E)-ω02(ϵrϵ0μrμ0-ιμrμ0σω0)E=0 in Ω,3n×E=0 on δΩ,4μ0μrϵ0ϵr-ισ/ωn×H+E-(n⋅E)n=-Es+(n⋅Es)n on δΩcoil,

where E is the RF electric field in $V\;m^{-1}$ ; $E_{s}$ is the RF electric field on the surface of the domain in $V\;m^{-1}$ ; $H=B/(\mu_{0}\mu_{r})$ is the RF magnetic field strength; $\epsilon_{0}$ and $\epsilon_{r}$ are the free space and relative permittivity, respectively; $\mu_{0}$ and $\mu_{r}$ are the free space and relative permeability, respectively; σ is the conductivity of the coil's material; n is the normal vector; and ω is the RF angular frequency. The computational domain was meshed using tetrahedral elements. The coil was truncated using the impedance boundary condition available in COMSOL, with this the coil's domain being excluded from the computation. The frequency range for the calculation used was from 1 to 3 GHz , where the frequency was swept in steps of 0.5 GHz using an adaptive frequency sweep. The lumped port for RF excitation was connected between the coil's terminal port, where the port's resistance was set to be 50 Ω to minimize insertion losses. The self-resonance of the coil was calculated using the reactance of the coil, where the frequency at which the reactance was zero was considered to be the self-resonance frequency. The RF efficiency was calculated by the following formula:

5 $$\text{RF efficiency}=\frac{B_{1}}{\sqrt{P}},$$

where $B_{1}$ is the total induced magnetic field in the sample volume in T , and P is the RF power applied in W . For the calculation, $B_{1}$ was calculated to be 45 MHz , which is the Larmor frequency of ${}^{1}H$ in a magnetic field of 1.05 T . The results are summarized in Table [Table T1].

**Table 1 T1:** Different coils simulated, their self-resonance, and RF efficiency calculated by means of the averaged $B_{1}$ field of the sample region for 1 W of power applied for ${}^{1}H$ at 45 MHz . A cylindrical sample geometry with a diameter of 1 mm and a height of 1 mm was taken as a standard for all the coil geometries.

Coil	Turn	Pitch	Self-resonance	RF efficiency
no.	no.	( mm )	( GHz )	( $\mathrm{kHz}\;{\sqrt{W}}^{-1}$ )
				(( $µT\;{\sqrt{W}}^{-1}$ ))
1	5	0.300	2.06	13.11 (307.9)
2	6	0.300	1.83	14.84 (348.5)
3	7	0.300	1.65	16.29 (382.53)
4	5	0.525	2.62	10.72 (251.91)
5	6	0.525	2.27	11.62 (273.05)
6	7	0.525	2.02	12.29 (288.62)
7	5	0.750	2.93	8.75 (205.44)
8	6	0.750	2.55	9.21 (216.38)
9	7	0.750	2.24	9.51 (222.34)

**Figure 1 F1:**
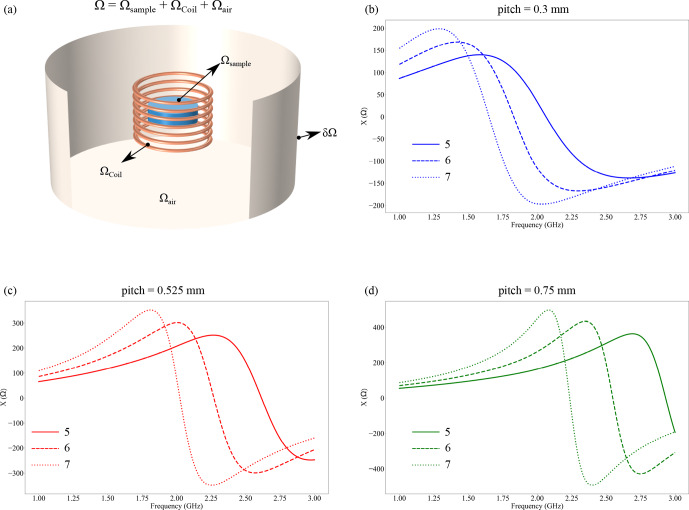
**(a)** Computational setup with different domains. The computational domain ( Ω ) was truncated by a perfect electric boundary condition. The lumped port was connected to the coil's terminal, where the S parameters were calculated to be in the range of 1–3 GHz , and the magnetic field produced by the coil was set at 45 MHz , i.e., the Larmor frequency of ${}^{1}H$ at 1.05 T . **(b–d)** Reactance of the coil simulated from the S parameters, with different pitch p and number of turns N . The frequency at which the reactance is zero is reported as the coil's self-resonance.

The S parameters generated from the electromagnetic simulation were exported as Touchstone files (S1P) and used in the circuit simulation to determine the effective self-resonance and quality factor of the coil when mounted on a printed circuit board (PCB). For the circuit simulation, Advanced Design System 2015.01 from Keysight Technologies was used. The PCB onto which the coil was to be mounted for measurement on the vector network analyzer (VNA) was modeled as shown in Fig. [Fig F2]a, where the effect of the PCB on the coil parameters was determined by measuring the impedance between the ports, with the port's impedance being set to 50 Ω to minimize insertion losses. The influence of the conductive adhesive used to connect the printed coil to the PCB is probably the biggest source of variance in the setup. However, since it is an unknown variation, it was neglected in the simulation, and an idealized value was used. Similarly to the method above, the frequency where the reactance was zero is reported as the self-resonance of the coil. The quality factor Q of the coil was calculated at 45 MHz using the following formula:

6 $$Q=\frac{\omega L}{R},$$

where, in the numerator, ωL is the reactance of the coil, such that ω is the angular frequency, and L is the inductance, and, in the denominator, R is the resistance at 45 MHz . The results for various coil geometries are summarized in Table [Table T2].

**Table 2 T2:** The Q factor, self-resonance, inductance, and resistance of the electrical circuit, with the latter data being compared for simulation and measurement results (in brackets). The coil is placed directly on the PCB shown in the Fig. [Fig F2]a. The self-resonances without the PCB are the simulation results from Table [Table T1].

Coil	Q factor	Self-resonance (with PCB)	Self-resonance (w/o PCB)	Inductance	Resistance
no.	(.)	( MHz ) $\left(\left(\mathrm{MHz}\right)\right)$	(GHz)	nH $\left(\mathrm{nH}\right)$	Ω $\left(\Omega \right)$
1	2.907 (3.156)	685 (540)	2.06	287.2 (49.18)	27.93 (4.42)
2	2.614 (4.035)	548 (520)	1.83	388.7 (56.57)	42.04 (3.98)
3	2.306 (4.172)	434 (480)	1.65	507.9 (64.24)	62.27 (4.37)
4	6.828( (2.874)	839 (670)	2.63	216.7 (42.28)	8.97 (4.17)
5	6.729 (3.295)	724 (630)	2.27	282.7 (47.26)	11.88 (4.07)
6	6.424 (3.443)	620 (600)	2.02	357.4 (52.31)	15.73 (4.31)
7	10.924 (2.788)	910 (680)	2.93	181.0 (39.13)	4.68 (3.98)
8	11.277 (3.152)	817 (660)	2.55	229.7 (42.71)	5.76 (3.84)
9	11.130 (3.353)	727 (640)	2.24	282.9 (39.70)	7.19 (3.97)

From the simulation results, it was found that the coil with a pitch p of 0.525 mm and five turns best fitted the requirements since it could be tuned and matched with standard fixed-valued discrete capacitors as our intention was to avoid the use of bulky non-magnetic trimmer capacitors. Moreover, it had a decent quality factor and was sensitive enough to be used as a transceiver coil.

**Figure 2 F2:**
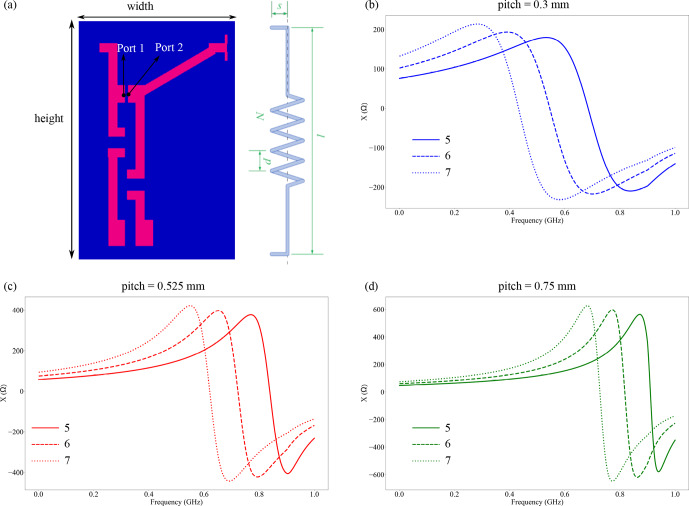
**(a)** The PCB used for the simulation, where the geometry was determined from the PCB which was used for the measurement from the VNA. The height of the PCB was 49 mm, and the width was 15 mm. Next to it is a schematic of a solenoidal coil. **(b–d)** Reactance of the coil mounted on the PCB shown in **(a)**, with different pitch p and number of turns N . The frequency at which the reactance is zero is reported as the setup's self-resonance.

The S1P files from the EM field simulations and the circuit simulations for all the coils are available within the dataset of this article, which can be used to determine the coil's characteristics at different frequencies.

## 3 Fabrication of the coil

The challenging part of fabricating MR micro-coils is the choice and design of the support structures for the coils since the material properties affect the static magnetic field [Bibr bib1.bibx9]. Different materials such as polyimide [Bibr bib1.bibx30], SU-8 [Bibr bib1.bibx26], and Teflon [Bibr bib1.bibx9] have been used as support structures. Existing techniques for fabricating micro-coils for NMR are using photolithographic methods, wire-bonding, rolling up of 2D printed structures, or by milling [Bibr bib1.bibx26]. Out of this list, micro-electro-mechanical system (MEMS)-like fabrication processes would be favorable due to their well-established high precision and repeatability. However, designing a coil directly on a cylindrical glass sample container (or on 3D structures) is complicated since available MEMS processes focus on planar substrates. Nevertheless, to fabricate 3D or complex structured coils, a fabrication technique based on extrusion printing can allow us to achieve the requirements [Bibr bib1.bibx29]. Extrusion printing allows us to extrude highly viscous, metal–nanoparticle inks, e.g., commercially available conductive screen-printing inks, through customized glass-printing nozzles for direct deposition on planar substrates and 3D parts such as glass capillaries [Bibr bib1.bibx29]. Hence, we combine the advantages of commercial screen-printing inks with those of digital printing processes to reproducibly fabricate micro-coils directly on glass tubes.

The extrusion-printing system applied consists of a granite table (A) carrying two linear stages (Owis LIMES 122-160-HSM) arranged crosswise on top of each other to form an xy stage, as shown in Fig. [Fig F3]. A gantry (B) consisting of Owis S65-4 system profiles is mounted on the table, which carries an additional linear stage (Owis LIMES 60-70-HSM) as the z axis of the Cartesian motion system. The printhead (C) is mounted onto stage of the z axis. For printing onto cylindrical substrates such as glass tubes, we developed an adapted, motorized dividing head that is mounted onto the xy stage. The dividing head consists of a goniometer stage (D) and a rotary stage (E) for manual alignment of the tube axis parallel to the y axis. Onto this setup are fixed the rotational axis (b), composed of a mechanical support, namely a Nanotec ST4118S1006-A stepper motor with Nanotec GPLL40-49; a flexible shaft coupling (F) for turning the substrate; and a bearing block with clamps (G) to mount cylindrical substrates. Furthermore, the EP system comprises two optical systems (H and I).

**Figure 3 F3:**
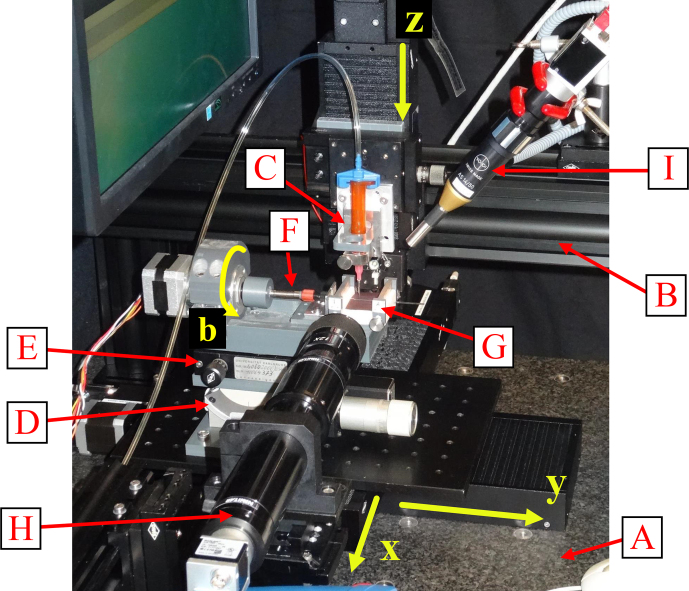
Extrusion-printing system for cylindrical substrates. The main components are the granite table (A), gantry (B), printhead (C), xybz stage, goniometer stage (D) with a rotary stage (E), flexible shaft coupling (F), bearing block (G), and optical systems (H) and (I).

The optical system (H) consists of a Basler acA1920-25uc color USB 3.0 camera and a Navitar 12× UltraZoom 12 mm fine-focus lens set (1-50504 Feinfokus 12× Objektiv, 1-6010 C-mount adapter, 1-6030 2× standard adapter, 2-50145 converter lens, 1-6270 universal mounting clamp) microscopic lens. Together with a Bivar L2-PGC1-F LED for transmitted light illumination, it is applied for manual alignment of the tube parallel to the y axis, as well as for control of the distance between the nozzle and substrate. The second optical system (I), composed of a Basler ac1600-20gm monochrome GigE camera and a Volpi AS 14/50 focusable borescope with a C-mount connector, light guide, and coaxial incident light illumination (Volpi intralux^®^ dc-1100), is used for manual alignment of the substrate relative to the printhead.

The printhead is composed of a Nordson 3 ${\mathrm{cm}}^{3}$ syringe barrel that is fixed on the z axis via a magnetic clamping setup, a printing nozzle, and a Nordson syringe barrel adapter that connects the cartridge to a Nordson Ultimus I time–pressure dispensing unit. As the control system, the Beckhoff basic CPU module CX1020-0123 with the software PLC TC1260 TwinCAT 2 is used in combination with EL7047 stepper motor terminals and EL1018/EL2008 digital input–output terminals. After manual setting of the local coordinate system of the PCB by means of a Logitech F310 joystick, the printing process is automated, controlled by the TwinCAT software.

The NMR micro-coils are printed onto borosilicate glass tubes with a length of 101.6 mm , an inner diameter of 1.0 mm , and an outer diameter of 1.5 mm (World Precision Instruments (WPI): PG52151-4). As conductive ink, the thixotropic screen-printing paste NPS from Harima, filled with silver nanoparticles (NPs) (nominal size 12 nm ), was selected. According to the certificate of analysis, the batch applied for the coil printing has a metal content of 82.3 wt% . A shear thinning behavior was confirmed by means of plate / plate rheometry measurements: about 510 Pas at a shear rate of 5 $s^{-1}$ and 44 Pas at 50.2 $s^{-1}$ [Bibr bib1.bibx29].

**Figure 4 F4:**
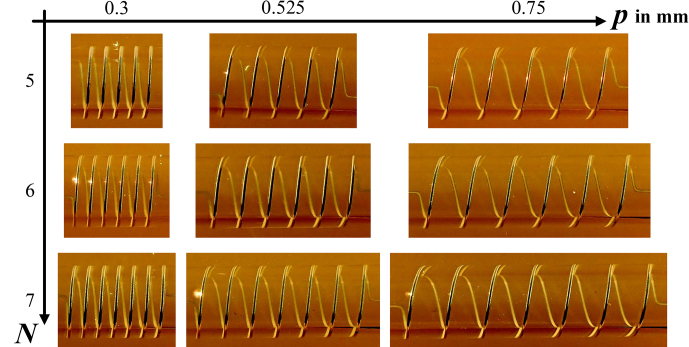
Extrusion-printed solenoid-coil variants on glass tubes.

The coil type to be realized was the solenoid coil as depicted in Fig. [Fig F2]a, where the parameters of a constant pitch p and the number of turns N were chosen from the geometry simulated, as shown in Table [Table T1]. The overall length l and the length s of the contact line end were based on the connection point on the PCB (as shown in Fig. [Fig F2]a) where the coil was to be mounted. Each coil variation presented in Table [Table T1] was printed three times in order to assess the reproducibility of the fabrication process in terms of the electrical properties of the coils.

As discovered in first printing tests, the glass tubes onto which the coils were to be printed showed variances in terms of geometric tolerances such as axial runout, straightness, and cylindricity. These variances resulted in varying distances between the nozzle and the cylindrical surface of the tube during printing. Neglecting the tolerances, reproducible printing without distance control between the nozzle and tube is not possible. As there will always be fabrication tolerances from the tube-manufacturing process, this issue was solved by means of optical inspection and sorting of the tubes prior to printing. Glass tubes with an overall radial deviation of more than 8 µm were rejected. Thus, a package of 100 tubes from WPI (PG52151-4) led to a rejection rate of about 22 % .

Following the methodology described by [Bibr bib1.bibx29], a glass capillary with $d_{i}=50.6\;µm$ as the printing nozzle was used for printing, with a pressure p in the range of 2.85 Pas to 2.98 bar (without back pressure $p_{\mathrm{vac}}$ ), a distance Δz=35µm between the nozzle and substrate, and a printing speed of $v_{p}=15\;\mathrm{mm}\;\min^{-1}$ .

After printing, the silver inks are dried, cured, and sintered in a Memmert UP 500 oven for 60 min at 220 °C and are then stored at room temperature. Silver-filled, epoxy-based conductive adhesive (Henkel Loctite 3880) is used for mounting the manufactured coils onto PCBs for electrical analysis and NMR measurements. Prior to mounting, the silver solenoid coils are surface treated for 60 s at 0.44 mbar and 15 W in a low-pressure argon plasma (Diener Atto plasma cleaner, with 13.56 MHz ) to remove silver oxides. The glass tubes with the solenoid coils are cut to length to match the width of the PCB. To mount the coils onto the PCB's contact pads, the tubes are manually aligned under a stereoscopic microscope. Conductive adhesive (Loctite 3880) is manually applied onto the PCB contact pads by means of a gauge-20 dispensing tip (Vieweg 505286, $d_{i}=580\;µm$ ) connected via a 3 ${\mathrm{cm}}^{3}$ syringe barrel and an adapter with tubing to a time–pressure dispenser (Nordson Ultimus I). The resulting bonds are cured in an oven for 8 min at 130 °C. The assembly was then visually inspected for printing defects through macroscopic images. Figure [Fig F4] gives an overview of the printed- and mounted-coil variants. For each variant, one of three realized coils is shown.

In addition to the visual inspection, the turns of a few coils are analyzed in terms of width, thickness, and cross-section by means of a Keyence VK-9700 confocal laser scanning microscope (LSM). Figure [Fig F5] shows the assembly of a coil, with p=0.3mm and N=5 , on a PCB (A) and a microscopic image of one of the adhesive joints (B) made of conductive adhesive between the PCB and the printed contact line. Furthermore, a microscopic image (C) and the LSM height profile (D) of a winding section are depicted. For this coil, the LSM measurements yield a structure width of 59.2 µm ; a thickness of 8.34 µm , resulting in an aspect ratio of 0.14 ; and a cross-section of 317 $µm^{2}$ . The aspect ratio of a printed line describes the quotient of layer thickness and width. This ratio should be as high as possible in order to achieve sufficient conductivity when miniaturizing the line width.

**Figure 5 F5:**
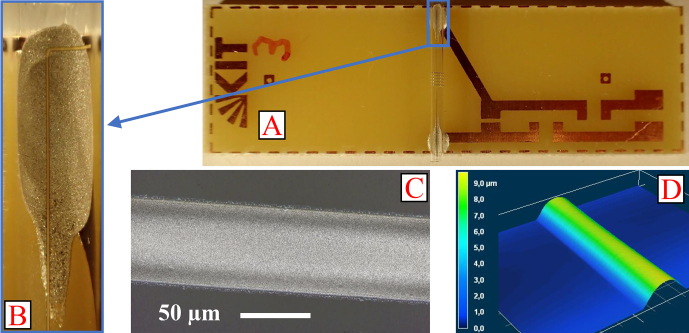
A solenoid coil with the parameters p=0.3 mm and N=5 mounted on a PCB (A), with a detailed image of the contact (B). Microscopic image (C) and LSM height profile (D) of a winding section.

Using an impedance analyzer (Agilent E4991A) and a probe station (Cascade Microtech MPS150), all of the realized NMR–coil–PCB assemblies are analyzed in terms of the parameters of resistance R , inductance L , modulus of the impedance |Z| , and quality factor Q over a measuring frequency range of 1–2000 MHz , with 801 points. The resistance values were measured at a frequency of 4.98 MHz , which was an arbitrarily chosen value as it was far away from the ${}^{1}H$ frequency used in the measurement, and the resistance values were similar to the DC resistance.

Both impedance analysis and LSM inspection show the high reproducibility of the realized coils and assemblies. Figure [Fig F6] substantiates this statement, showing the reactance of various samples of two coil parameter sets. The progression of the measurement curves for all three coils of a parameter set is almost identical. These properties are the same for the measured resistance and modulus of impedance as well.

**Figure 6 F6:**
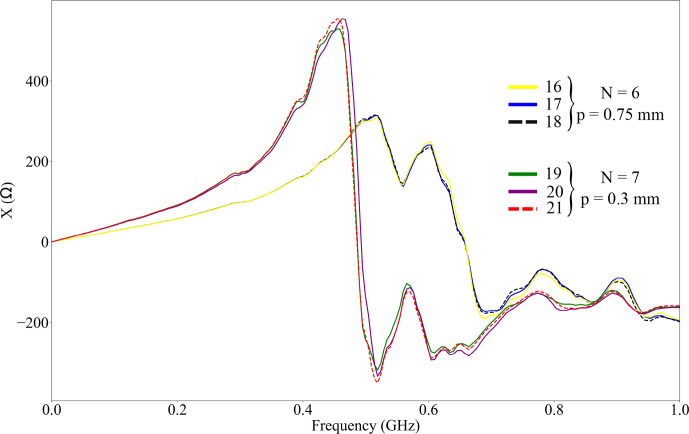
Coils' reactance X over frequency f of three solenoid coils with N=6 and p=0.75mm and of three coils with N=7 and p=0.3mm .

This reproducibility is only true for the extrusion-printed-coil structures. Conductive adhesive (Loctite 3880) is used to connect the copper tracks to the PCB. To estimate the impedance of these adhesive contacts, three PCBs on which no coils but rods of copper wire (diameter 1.08 mm ) are mounted were measured. At 4.98 MHz , the resistance has a mean of 0.0701 Ω and a standard deviation of 0.0398 Ω . The high standard deviation reflects the poor reproducibility of manual adhesive dispensing and coil mounting. Nevertheless, these results show that the resistance of the conductive adhesive joints can be neglected for the realized coils. Based on microscopic measurements and calculations, the total conductive length of some solenoid coils between both adhesive joints is determined. In combination with the LSM profile measurements, the resistivity of one of three coils with N=5 and p=0.3mm is calculated as 3.96 µΩcm . This value is obtained from the coil's serial resistance of 4.17 Ω at 4.98 MHz minus the mean value of the copper rod assembly and corresponds to about 2.5 times the resistivity of bulk silver at 298 K ([Bibr bib1.bibx18], pdf p. 2123 f. (12–42 ff.)).

## 4 Proton magnetic resonance spectroscopy and imaging at 1.05 T

The NMR spectrum and images were acquired in a horizontal-bore 1.05 T imaging magnet (ICON, Bruker). The coil had a pitch p of 0.525 mm , with five turns. The combined coil and capillary were mounted on the PCB, as shown in Fig. [Fig F7]a, which was different from what was used for the simulation of the S parameter and for the characterization of the coil, as shown in Fig. [Fig F5]a. The PCB was different due to the magnetic field direction and restricted space inside the magnet. Nevertheless, the coil was also tuned to 45 MHz and matched to 50 Ω .

For acquiring the NMR spectrum, the glass capillary supporting the extrusion-printed coil was filled with pure ethanol. The excitation power applied to the coil was 0.1 W , with a π/2 pulse duration of 27 µs and a receiver gain of 203. The spectrum was acquired with a single scan. It is plotted in Fig. [Fig F7]b using the Python library nmrglue (release 0.9-dev, date: 5 December 2021). From the spectrum, the RF efficiency measured was 29.28 $\mathrm{KHz}{\sqrt{W}}^{-1}$ , and the SNR of the triplet at around 1.3 ppm was 538.65.

**Figure 7 F7:**
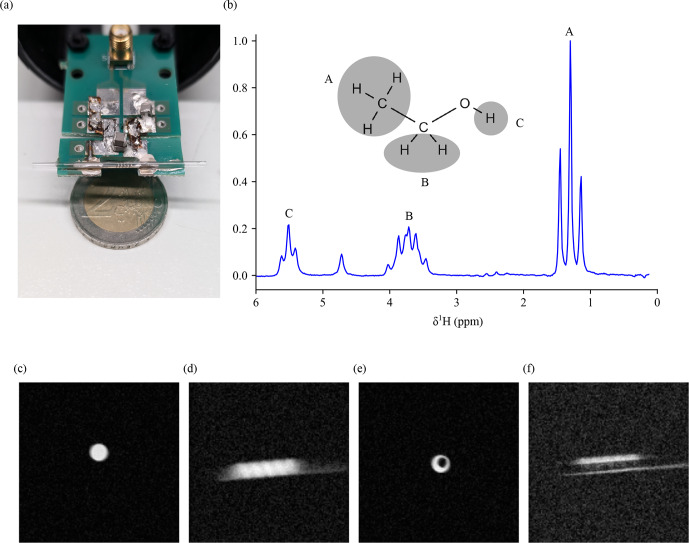
**(a)** The glass capillary with the printed coil used for the acquisition with the sample inside. **(b)** Homonuclear ${}^{1}H$ NMR spectrum of pure ethanol acquired in a 1.05 T imaging magnet (RF excitation power of 0.1 W , π/2 -pulse duration of 27 µs ). **(c–f)** MR images with the coil. Panels **(c)** and **(d)**, with a completely filled capillary, show a sagittal and an axial image, respectively. Panels **(e)** and **(f)** also shows a sagittal and an axial image but with a Teflon tube with a 0.5 mm outer diameter placed in the capillary and with the sample filled around it. Since Teflon ( $C_{2}F_{4}$ ) has no ${}^{1}H$ nuclei, it has no ${}^{1}H$ NMR signal. This creates a defined border and highlights the spatial resolution achievable.

The MR images acquired are shown in Fig. [Fig F7]c–f. In order, the images acquired were (c) a sagittal image and (d) an axial image of a capillary completely filled with distilled water. Similarly, panel (e) shows a sagittal image and panel (f) shows an axial image of a capillary filled with distilled water around an unfilled Teflon tube with an outer diameter of 0.5 mm . While panels (c) and (d) show the signal-to-noise ratio of the coil, the Teflon tube in (e) and (f) was used to provide a recognizable structure that can highlight the achievable spatial resolution.

For image acquisition, the pulse sequence used was a gradient echo sequence with an echo time (TE) of 4 ms , a repetition time (TR) of 100 ms , a flip angle (FA) of 30°, a slice thickness of 2 mm , an image size of 128 px × 128 px, and a 10 mm × 10 mm field of view (FOV) resulting in an in-plane resolution of 78 µm . The number of scans was 32 for sagittal images and 64 for axial images. The signal-to-noise ratio (SNR) values of the MR images are 68, 36, 58, and 17.

## 5 Conclusion

In this article, we demonstrated a method for rapidly prototyping coils using modeling, simulation, and additive manufacturing. The geometry of the coils was computed for their efficiency, i.e., the magnetic fields generated for the power applied to the coils, and their self-resonance. From these computations, the reflection parameters were generated, which were used in the simulation of the printed circuit boards on which the coils were to be mounted. This helped to provide the effective self-resonance of the system, which was significantly lower than the actual computed values. Therefore, the coil which had a decent quality factor and RF efficiency and could be tuned to the Larmor frequency of ${}^{1}H$ was chosen for the fabrication.

The coils were additively manufactured by extrusion printing on glass capillaries and were tested in a 1.05 T horizontal-bore imaging magnet. The spectrum of pure ethanol was recorded, where the coil could distinguish J coupling even at low field values. The splitting shown in the spectrum due to J coupling shows the spectral quality of the coils. Similarly, the MRI results demonstrate the high sensitivity of the printed coil, enabling high-resolution imaging with decent SNR.

These results show the NMR compatibility of the coils, which can now be rapidly produced with a low-iteration process tailored to the application of both spectroscopy and imaging. Solenoids were chosen in this study because this type allows us to clearly demonstrate the effects of design parameter variation and repeatability.

In future studies, we also want to apply this modeling, simulation, and extrusion-printing workflow to investigate other more general coil classes like birdcage or butterfly coils that are otherwise very complicated to fabricate.

The limitations of the extrusion-printing process in terms of resolution and of the line width are mainly determined by the properties of the ink in terms of particle size and rheology, especially regarding its thixotropic behavior. The line width can be reduced by reducing the nozzle orifice. This also affects the distance between the nozzle and the substrate during printing. This distance can be approximated to be 0.5 times the inner diameter of the nozzle orifice. For very small nozzle orifices, precise automatic control of the distance between the nozzle and substrate is the key. Measuring sub-micron deviations in the stand-off distance when rotating the glass tubes to be printed on is challenging and had its limitations with regard to the measurement equipment being available when the device was built. Printing line widths in the range of hundreds of microns was possible, but reducing the line width further would have led to a reduced conductor's cross-section and, hence, a decrease in the conductivity of the printed tracks. In addition, the tracks need to withstand a certain current. This also limits the miniaturization of the printed conductor. The pitch of the coils can be reduced to the point where the windings tend to touch when the pitch is equal to the line width. For even smaller pitches, a multilayer printing approach is required, with additional isolating layers. Alternatively, the line width can be increased at a constant pitch until the tracks tend to touch. The line thickness depends strongly on the rheology of the ink. Printing parameters and the surface of the glass only have minor influences on the thickness. To increase the thickness of the tracks, the rheology must be adjusted towards higher viscosity and a certain thixotropic of the behavior inks. For higher aspect ratios, multi-pass printing instead of single-pass printing can be a solution, but this can affect the track's edge quality. The main issue when changing the outer diameter of the coil is an appropriate mounting of the tube with the changed diameter. Larger variations in tube diameter due to tube fabrication tolerances probably require a revision of the setup for mounting the tubes. Certainly, a diameter of 150 µm will be challenging and probably not compatible with the bearing blocks of the presented system but feasible with a modification of the setup.

A future improvement in the extrusion-printing equipment lies in the implementation of a closed-loop control of the distance between the printing nozzle and the sample container. This would reduce the requirements of low-dimensional tolerances of commercial glass tubes and would thereby further simplify the process. Moreover, we intend to improve the mounting of the printed coils onto the PCBs and also include it in the modeling and simulation to even more precisely forecast printed-coil performance. The flexibility of the printing system allows for complicated shapes and precision, which can now be directly fabricated on the sample container. The coils produced with our method show such small sample-to-sample variation that the tuning and matching of the samples become very easy and could, for example, be done with a low-cost tuning and matching circuit [Bibr bib1.bibx14].

## Data Availability

The measurement and simulation data can be found in a KITOpen repository under 10.35097/8eeh3psqj9a0vwx5 [Bibr bib1.bibx19].
